# The Combination of Physiological and Transcriptomic Approaches Reveals New Insights into the Molecular Mechanisms of *Leymus chinensis* Growth Under Different Shading Intensities

**DOI:** 10.3390/ijms26062730

**Published:** 2025-03-18

**Authors:** Xinru Li, Qianqian Yu, Zhongxu Yao, Shuo Li, Lichao Ma, Kunlong Su, Guofeng Yang

**Affiliations:** 1 College of Grassland Science, Qingdao Agricultural University, Qingdao 266109, China; lixinru0404@163.com (X.L.); 18389166388@163.com (Q.Y.); 17660433151@163.com (Z.Y.); 2 Key Laboratory of National Forestry and Grassland Administration on Grassland Resources and Ecology in the Yellow River Delta, Qingdao 266109, China; lishuo@qau.edu.cn (S.L.); ma-lichao@163.com (L.M.); 3 Agricultural Research Institute of Saline and Alkaline Land of Yellow River Delta, Dongying 257000, China

**Keywords:** shade stress, *Leymus chinensis*, transcriptome, physiology, growth and development

## Abstract

*Leymus chinensis* is a grass species in the family Triticeae that is found in the Eurasian grassland region and is known for its outstanding ecological advantages and economic value. However, the increasing adoption of photovoltaic agriculture has modified the light environment for the grass, markedly inhibiting its photosynthesis, growth, and yield. This study used physiological and transcriptomic analyses to investigate the complex response mechanisms of two *L. chinensis* genotypes (Zhongke No. 3 [*Lc3*] and Zhongke No. 5 [*Lc5*]) under shading stress. Growth phenotype analysis revealed the superior growth performance of *Lc3* under shading stress, evidenced by enhanced plant height and photosynthetic parameters. Additionally, differentially expressed genes (DEGs) were predominantly enriched in starch and sucrose metabolism and glycolysis/gluconeogenesis pathways, which were the most consistently enriched in both *L. chinensis* genotypes. However, the flavonoid biosynthesis and galactose metabolism pathways were more enriched in *Lc3*. Weighted gene co-expression network analysis identified the *LcGolS2* gene, which encodes galactinol synthase, as a potential hub gene for resistance to shade stress in comparisons across different cultivars and shading treatments. The use of qRT-PCR analysis further validated the genes involved in these pathways, suggesting that they may play critical roles in regulating the growth and development of *L. chinensis* under shading conditions. These findings provide new insights into the molecular mechanisms underlying the growth and development of *L. chinensis* under different shading stress conditions.

## 1. Introduction

*Leymus chinensis* (Trin. ex Bunge) Tzvelev serves as a key species in the grassland ecosystems of the Songnen Plain in northeastern China and the eastern Inner Mongolia grasslands. The grass also holds a crucial position in the meadow and dry steppes of eastern Eurasia [1]. This forage grass is widely used in forage production due to its well-developed rhizomes, lush foliage, high yield, and rich protein content [2]. Its remarkable adaptability to cold, salinity, and drought has also made it an important species in livestock farming and sustainable agriculture [3,4,5,6,7]. However, as photovoltaic installations become more widespread, the shading conditions under solar panels significantly modify the light environment, restricting the photosynthesis and growth performance of *L. chinensis* and leading to reduced productivity [8]. This shift in the light environment directly challenges the plant’s physiological processes, making it essential to investigate the growth changes of *L. chinensis* under shading stress. Therefore, understanding its adaptive mechanisms and enhancing its stress tolerance through modern molecular breeding techniques can help improve its yield under shaded conditions.

Light is a fundamental environmental factor influencing plant growth and development because its intensity and quality regulate photosynthesis, growth patterns, and metabolic processes in leaves [9,10]. Reduced light intensity that results from shading triggers significant physiological and morphological changes in plants, including alterations in polysaccharide biosynthesis, secondary metabolite accumulation, and overall metabolic activity [11,12]. This stress disrupts key processes such as photosynthesis, gas exchange, and antioxidant activity, ultimately impairing the normal growth of plants [13,14,15]. One of the most prominent consequences of shading is the overaccumulation of reactive oxygen species (ROS), which leads to oxidative stress and cellular damage. Excess ROS can damage cellular components, including lipid membranes, proteins, and DNA, and also affect biomarkers such as malondialdehyde (MDA) and superoxide dismutase (SOD) [16,17]. This stress triggers physiological changes, including the activation of plant defense mechanisms aimed at mitigating oxidative damage. To combat ROS-induced stress, plants synthesize non-enzymatic antioxidants like flavonoids, carotenoids, ascorbic acid (AsA), and glutathione (GSH) through robust antioxidant systems. Concurrently, plants also activate enzymatic antioxidants such as SOD, peroxidase (POD), and catalase (CAT) [18,19]. Among these, flavonoids have been identified as particularly effective non-enzymatic antioxidants and play a critical role in alleviating oxidative damage caused by shading stress [20].

At the molecular level, plants adapt to shading stress through intricate gene regulatory networks. For example, *AtBBX24*, *AtPIF4*, and *AtDELLA* genes significantly enhance shade tolerance by regulating signaling pathways and stress response mechanisms in *Arabidopsis* [21]. Similarly, the *PIF4* gene serves as a key regulatory factor for rapid growth under shading conditions in tomatoes by encoding proteins involved in auxin synthesis and signal transduction [22]. In addition, the *GolS* gene plays a critical role in the adaptation of *Ajuga reptans* to shading stress by regulating the synthesis of raffinose family oligosaccharides under shaded conditions [23]. This regulation influences key physiological processes, including photosynthesis and signaling pathways, highlighting its importance in enhancing plant resilience to low-light environments.

The rapid growth of photovoltaic agriculture has increased the complexity of shading environments for plants [24]. As a key species for ecological restoration, *L. chinensis* encounters several environmental constraints that significantly impact its growth under shading stress [25]. At the physiological level, shading stress in *L. chinensis* induces notable changes, including the downregulation of photosynthesis-related genes and the upregulation of sugar metabolism genes, which facilitate energy redistribution. Additionally, hormone-mediated mechanisms promote leaf elongation and cell expansion, enabling the plants to adapt to reduced light availability [3,26]. The plants also accumulate secondary metabolites as a potential strategy for adapting to shade. Despite these insights into physiological changes that occur under shading stress, the molecular mechanisms underlying shade tolerance in *L. chinensis* remain largely unexplored. The current research predominantly focuses on ecological and physiological aspects, with limited studies investigating gene regulatory networks or metabolic pathways specific to *L. chinensis*. Comparative studies have shown significant variation among the soybean, peanut, and sweet potato germplasms under shading stress [15,20,27]; for *L. chinensis*, similar investigations are scarce. Nevertheless, understanding these molecular mechanisms could provide critical insights for enhancing the ecological benefits and economic potential of *L. chinensis* in shaded environments, and lay a foundation for the sustainable development of the forage industry.

Additionally, transcriptomics has emerged as a powerful approach for uncovering gene functions and biological mechanisms, particularly in plant stress responses. Its technology has been extensively utilized to study shading stress in crops such as foxtail millet, sweet potato, and soybean [20,28,29]. For instance, transcriptomic analyses of foxtail millet cultivars with differing shade tolerances have revealed key contributors to shade tolerance. These include starch and sucrose metabolism, as well as carotenoid biosynthesis pathways. Differences in shade tolerance were also observed between the shade-tolerant *Lc3* and shade-intolerant *Lc5 L. chinensis* cultivars. These findings help to systematically elucidate the molecular mechanisms involved in shading stress. These findings will not only offer valuable insights into the molecular mechanisms involved in shade tolerance in *L. chinensis* but also provide new perspectives for optimizing its cultivation practices, enhancing its ecological and economic benefits in photovoltaic agricultural systems, and promoting grassland ecological restoration.

## 2. Results

### 2.1. Growth Performance, Chlorophyll Content, and Photosynthesis

Preliminary shading experiments were conducted on six *L. chinensis* cultivars—*Lc1*, *Lc2*, *Lc3*, *Lc5*, *Lc7*, and *Lc9*—using shading nets with varying densities (S0, S1, and S2). After 30 days of shading, significant variations in growth performance were observed in S2-treated plants. Subsequent initial growth parameter measurements indicated that *Lc3* plants demonstrated exceptional growth performance, while *Lc5* exhibited comparatively weaker growth performance. Based on these findings, *Lc3* and *Lc5* cultivars were selected for further physiological and transcriptomic analyses under three shading conditions: S0 (natural light), S1 (single-layer shading), and S2 (double-layer shading).

**Figure 1:**
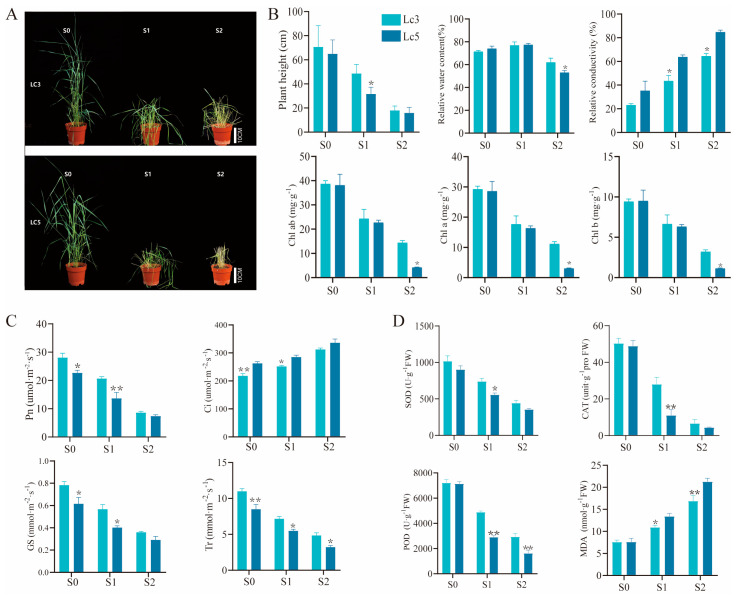
Effects of shade stress on phenotypic and physiological indices of *L. chinensis*. (**A**) Zhongke No. 3 (*Lc3*) and Zhongke No. 5 (*Lc5*) phenotypes during the treatment period. Scale bar  =  10 cm. (**B**) The plant height, relative water content, relative electrical conductivity, and chlorophyll content. (**C**) The gas exchange parameters. (**D**) The impact of varying levels of shading on the activities of superoxide dismutase (SOD), peroxidase (POD), and catalase (CAT) and the content of malondialdehyde (MDA) in the leaves of *Leymus chinensis* plants. “*” and “**” indicate a significant difference (*p* < 0.05) and an extremely significant difference (*p* < 0.01) between *Lc3* and *Lc5*, respectively.

As shading intensity increased, *Lc3* and *Lc5* cultivars exhibited distinct morphological differences (Figure 1A). With S1, the plant height of *Lc3* was significantly greater than that of *Lc5* (*p* < 0.05) (Figure 1B). With S2, *Lc3* showed significantly higher relative water content and levels of chlorophyll a, chlorophyll b, and total chlorophyll than *Lc5* (*p* < 0.05). In contrast, the relative conductivity of *Lc3* was significantly lower than that of *Lc5* under both S1 and S2 treatments (*p* < 0.05).

Similarly, as the shading intensity increased, the net photosynthetic rate (Pn), stomatal conductance (Gs), and transpiration rate (Tr) exhibited a gradual decline, whereas the intercellular CO_2_ concentration (Ci) increased. Under the S1 treatment, the Pn, Gs, and Tr of *Lc5* were significantly reduced by 33.8%, 29.1%, and 23.4%, respectively, compared to *Lc3*. Conversely, the Ci of *Lc5* under the S1 treatment was significantly higher than that of *Lc3*, with an increase of 13.2%. As the shading intensity increased, the activities of SOD, CAT, and POD enzymes in *Lc3* and *Lc5* were significantly reduced (*p* < 0.05). Specifically, under the S1 treatment, the activities of SOD, CAT, and POD in *Lc3* were 32.9%, 154.0%, and 68.0% higher, respectively, than in *Lc5*. Under the S2 treatment, these enzymes’ activities in *Lc3* were 25.2%, 50.5%, and 80.1% higher, respectively, than in *Lc5*. Meanwhile, the MDA content significantly increased in both cultivars (*p* < 0.05). Compared to *Lc3*, the MDA content in *Lc5* increased by 22.6% and 26.1%, respectively, under the S1 and S2 treatments.

### 2.2. Transcriptome Analysis

The *Lc3* and *Lc5* cultivars exhibited significant differences in growth performance and physiological indicators under varying shading stresses, with *Lc3* demonstrating superior adaptability to shading stress compared to *Lc5*. Therefore, RNA sequencing was used to uncover the differences in gene expression between *Lc3* and *Lc5* cultivars under different shading stresses and to elucidate the molecular mechanisms underlying their varying adaptive capacities.

Eighteen cDNA libraries were constructed from *Lc3* and *Lc5* under three shading conditions (S0, S1, and S2) for transcriptomic analysis. After removing low-quality reads, an average of 6.63 Gb of clean reads was obtained. Sequencing quality was high, with Q30 and GC content percentages ranging from 96.67 to 95.14% and 54.66 to 51.26%, respectively, with a base error rate of 0.01% (Supplementary Table S1). The mapping and unique mapping rates exceeded 75.42% (Supplementary Table S2), confirming the suitability of the selected reference genome, and the principal component analysis (PCA) revealed significant differences in unigene expression between shading treatments (Supplementary Figure S1).

**Figure 2:**
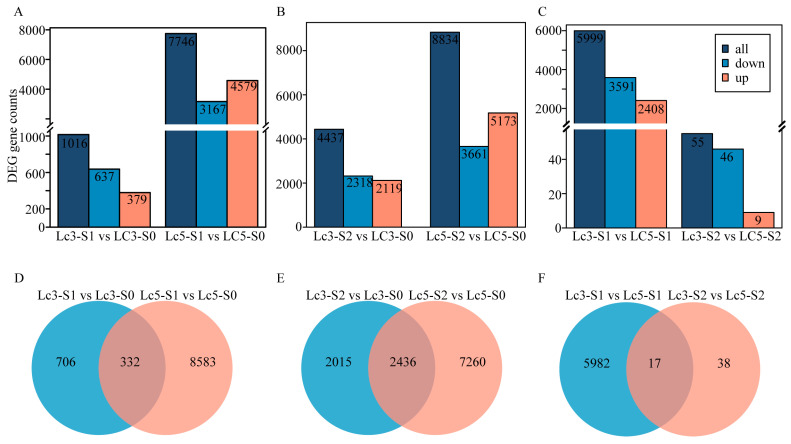
The profiles of DEGs related to the growth and development of two *L. chinensis* cultivars under shading stress. The number of upregulated and downregulated DEGs in the paired comparisons during the growth of *L. chinensis* under single-layer net (S1) (**A**) and double-layer net (S2) (**B**) shading stress. The Venn diagram of DEGs in paired comparisons under single-layer net (**D**) and double-layer net (**E**) shading stress. (**C**) The number of upregulated and downregulated DEGs in paired comparisons between different cultivars of *L. chinensis* under single-layer net and double-layer net shading. (**F**) The Venn diagram of DEGs in paired comparisons between different *L. chinensis* cultivars under single-layer and double-layer net shading.

The differential gene expression in *Lc3* and *Lc5* cultivars was analyzed by comparing the results of growth under S1 and S2 treatments to those under normal light conditions (S0). Under the S1 treatment, 1016 differentially expressed genes (DEGs) were identified in the *Lc3*-S1 vs. *Lc3*-S0, while 7746 DEGs were identified in the *Lc5*-S1 vs. *Lc5*-S0 (Figure 2A). Of the 1016 DEGs in the *Lc3*-S1 vs. *Lc3*-S0, 379 were upregulated and 637 were downregulated, while in the *Lc5*-S1 vs. *Lc5*-S0, 4579 DEGs were upregulated and 3167 DEGs were downregulated (Figure 2A). Under the S2 treatment, 4437 DEGs were identified in the *Lc3*-S2 vs. *Lc3*-S0, while 8834 DEGs were identified in the *Lc5*-S2 vs. *Lc5*-S0 (Figure 2B). Of the 4437 DEGs in the *Lc3*-S2 vs. *Lc3*-S0, 2119 were upregulated and 2318 were downregulated, while among the 8834 DEGs in the *Lc5*-S2 vs. *Lc5*-S0, 5173 genes were upregulated and 3661 were downregulated (Figure 2B). Furthermore, a Venn diagram identified 6038 DEGs across the *Lc3* and *Lc5* cultivars in the S1 treatment, with 5982 specific to *Lc3*-S1 vs. *Lc5*-S1, 38 specific to *Lc3*-S2 vs. *Lc5*-S2, and 17 shared between *Lc3*-S1 vs. *Lc5*-S1 and *Lc3*-S2 vs. *Lc5*-S2 (Figure 2F).

A Venn diagram detected 9622 DEGs in the S1 treatment across the *Lc3* and *Lc5* cultivars, including 706 specific to *Lc3*-S1 vs. *Lc3*-S0, 8583 specific to *Lc5*-S1 vs. *Lc5*-S0, and 332 common to both *Lc3*-S1 vs. *Lc3*-S0 and *Lc5*-S1 vs. *Lc5*-S0 (Figure 2D). Under the S2 treatment, 11,712 DEGs were identified across *Lc3* and *Lc5* cultivars, with 2015 unique to *Lc3*-S2 vs. *Lc3*-S0, 7260 unique to *Lc5*-S2 vs. *Lc5*-S0, and 2436 shared between comparisons of *Lc3*-S2 vs. *Lc3*-S0 and *Lc5*-S2 vs. *Lc5*-S0 (Figure 2E). Overall, under the S1 treatment, the number of DEGs in the *Lc5* genotype was greater than in *Lc3* (Figure 2A,D), while under the S2 treatment, the number of shared DEGs between the two genotypes increased, with *Lc3* having fewer total and unique DEGs compared to *Lc5* (Figure 2B,E). Thus, shading stress caused differential expression changes in growth-related genes between the two *Lc3* and *Lc5* genotypes.

Additionally, the differential gene expression levels in the two *L. chinensis* cultivars under the S1 treatment were compared. Under different shading stresses with the S1 and S2 treatments, specific DEGs were detected in *Lc3* that may be related to its shade tolerance. Compared to *Lc5*, *Lc3* had 5999 DEGs under single-layer net shading conditions (*Lc3*-S1 vs. *Lc5*-S1), with 2408 upregulated and 3591 downregulated, and 55 DEGs under double-layer net shading conditions (*Lc3*-S2 vs. *Lc5*-S2), with 9 upregulated and 46 downregulated (Figure 2C). Of these DEGs, *Lc3* exhibited 5982 and 38 unique DEGs during S1 and S2 shading stress levels, respectively (Figure 2F, Supplementary Table S3). Thus, these findings indicate that the gene responses related to *L. chinensis* growth under shading stress differ between genotypes.

### 2.3. Gene Ontology (GO) Functional Enrichment Analysis

**Figure 3:**
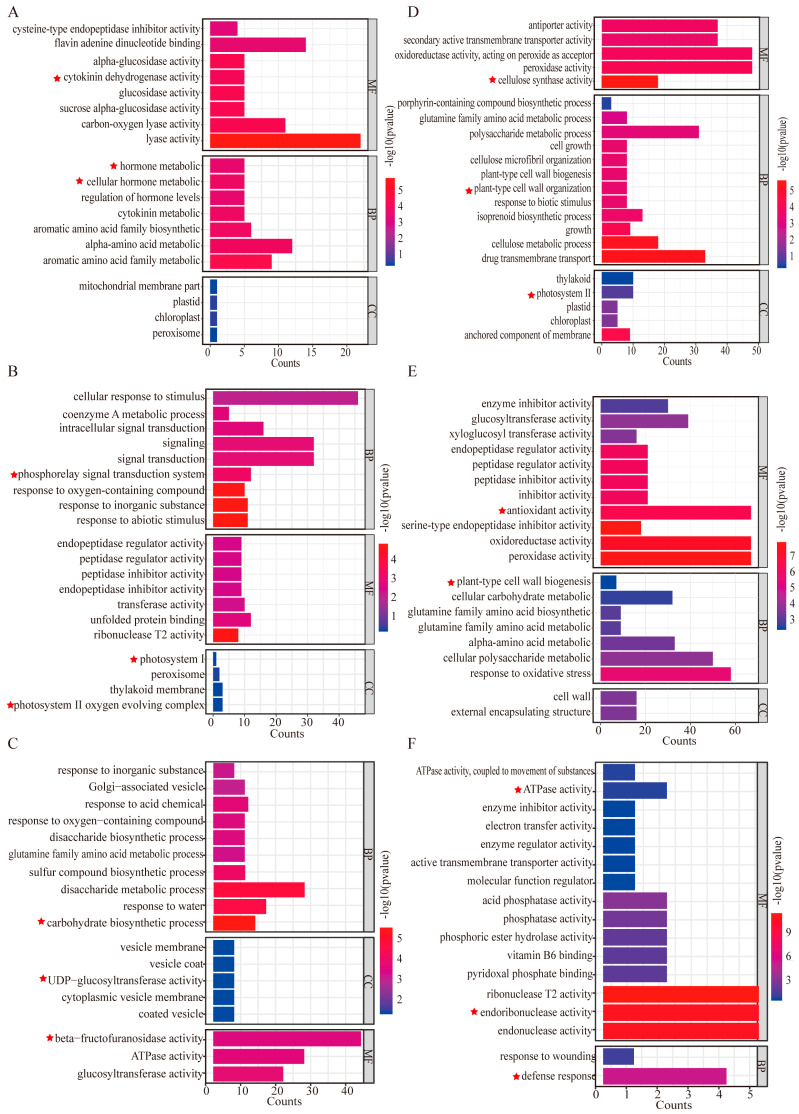
The top 20 enriched GO terms of the DEGs during the shading stress process in the two *Leymus chinensis* cultivars. (**A**) *Lc3*-S1 vs. *Lc3*-S0. (**B**) *Lc3*-S2 vs. *Lc3*-S0. (**C**) *Lc3*-S1 vs. *Lc5*-S1. (**D**) *Lc5*-S1 vs. *Lc5*-S0. (**E**) *Lc5*-S2 vs. *Lc5*-S0. (**F**) *Lc3*-S2 vs. *Lc5*-S2. S0: natural light, S1: single-layer shading, and S2: double-layer shading. *Lc3* and *Lc5* refer to Zhongke No. 3 and Zhongke No. 5, respectively.

The GO functional enrichment analysis revealed that the roles of DEGs involved in the growth and development of the two *L. chinensis* cultivars under shading stress by classifying them as either a biological process (BP), molecular function (MF), or cellular component (CC) (Figure 3, Supplementary Table S4). Thus, the DEGs in the comparison between *Lc3-S1* and *Lc3-S0* were significantly enriched in several GO terms, such as hormone metabolism, cellular hormone metabolism, and cytokinin dehydrogenase activity (Figure 3A). In contrast, the DEGs in the comparison between *Lc3-S2* and *Lc3-S0* were enriched in functions related to photosynthesis, including photosystem I and the photosystem II oxygen-evolving complex, and the phosphorelay signal transduction system (Figure 3B). These results suggest that *Lc3* relies on hormonal regulation and enhanced photosynthesis efficiency to mitigate shading stress. However, in *Lc5*-S1 vs. *Lc5*-S0, DEGs were enriched in structural and metabolic pathways, such as photosystem II, plant-type cell wall biogenesis, and cellulose synthase activity (Figure 3D), and in *Lc5*-S2 vs. *Lc5*-S0, plant-type cell wall biogenesis, antioxidant activity, and cellular carbohydrate metabolic processes were enriched (Figure 3E). These findings indicate that *Lc5* primarily employs structural and metabolic adaptations to cope with shading stress.

In *Lc3*-S1 vs. *Lc5*-S1, DEGs were predominantly enriched in the carbohydrate biosynthetic process, UDP-glucosyltransferase activity, and beta-fructofuranosidase activity (Figure 3C), suggesting metabolic divergence between the genotypes, while in the *Lc3*-S2 vs. *Lc5*-S2 comparison, DEGs were enriched in endoribonuclease activity, ATPase activity, and defense response (Figure 3F), highlighting genotype-specific stress response mechanisms. Overall, these results indicate that *Lc3* leaves enhance pathways such as carbohydrate biosynthetic processes and photosystem I to improve tolerance to shading stress, while *Lc5* emphasizes structural adaptations like cell wall biogenesis and antioxidant activities. These partially overlapping but distinct enrichment patterns likely account for the differences in growth and stress tolerance observed between the two cultivars, suggesting the need for further research into stress-resilient agronomic traits in grasses.

### 2.4. KEGG Enrichment Analysis of DEGs

**Figure 4:**
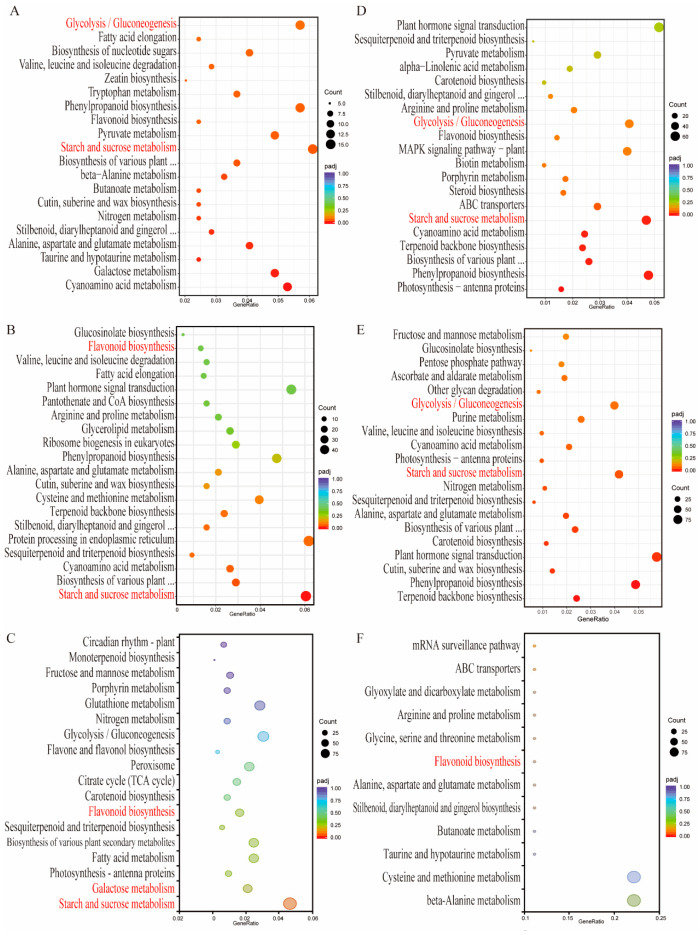
The top 20 KEGG-rich pathways of the DEGs during the growth of the two *L. chinensis* cultivars under different shade stresses. (**A**) *Lc3*-S1 vs. *Lc3*-S0. (**B**) *Lc3*-S2 vs. *Lc3*-S0. (**C**) *Lc3*-S1 vs. *Lc5*-S1. (**D**) *Lc5*-S1 vs. *Lc5*-S0. (**E**) *Lc5*-S2 vs. *Lc5*-S0. (**F**) *Lc3*-S2 vs. *Lc5*-S2.

A KEGG enrichment analysis of the DEGs was conducted to explore the molecular mechanisms underlying the growth and development of *Lc3* and *Lc5* genotypes under shading stress. In *Lc3*, 90 and 127 KEGG pathways were enriched under S1 (*Lc3*-S1 vs. *Lc3*-S0) and S2 (*Lc3*-S2 vs. *Lc3*-S0), respectively, while in *Lc5*, 130 and 129 pathways were enriched in *Lc5*-S1 vs. *Lc5*-S0 and *Lc5*-S2 vs. *Lc5*-S0, respectively. Additionally, 119 pathways were enriched in *Lc3*-S1 vs. *Lc5*-S1, while only 12 pathways were enriched in the *Lc3*-S2 vs. *Lc5*-S2 comparison (Supplementary Table S5). Among the top 20 pathways (Figure 4), starch and sucrose metabolism and glycolysis/gluconeogenesis were commonly enriched in all the comparison groups, suggesting a central role for carbohydrate metabolism and energy production in the growth and development response of *L. chinensis* to shading stress, regardless of shading intensity or genotype. The KEGG enrichment analysis of the two varieties (*Lc3*-S1 vs. *Lc5*-S1) revealed that the starch and sucrose metabolism, flavonoid biosynthesis, and galactose metabolism pathways were enriched in the S1 treatment of the shade-tolerant cultivar (Figure 4C). The flavonoid biosynthesis pathway was also enriched in the S2 treatment comparison between *Lc3*-S2 and *Lc5*-S2 (Figure 4F). These findings suggest that the DEGs involved in these pathways, along with their regulatory metabolic processes, may play a critical role in making *Lc3* more effective than *Lc5* with regard to shade stress and growth and development.

### 2.5. Weighted Gene Co-Expression Network Analysis (WGCNA)

**Figure 5:**
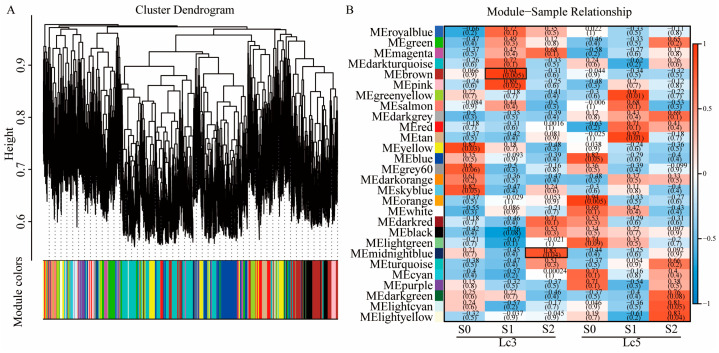
WGCNA of the growth and development of the two *Lc3* and *Lc5* cultivars under shading stress. (**A**) The hierarchical clustering tree shows the co-expression modules identified by WGCNA. (**B**) The correlation between samples and WGCNA co-expression modules, i.e., the relationship between modules (left) and traits (bottom). The red and blue backgrounds represent positive and negative correlations, respectively, with correlation coefficients and *p*-values provided.

To uncover the gene regulatory networks influencing the growth and development of *L. chinensis* under shading stress, we employed WGCNA using RNA sequencing data. Prior to network construction, sample clustering was performed to detect potential outliers. The dendrogram showed a clear separation of biological replicates, confirming the data’s quality and suitability for WGCNA (Supplementary Figure S2). Before constructing the gene co-expression network, the value of the soft threshold power β was calculated, and the scale-free topology threshold of the network was set at 0.6. When the soft threshold power was 10, the average connectivity approached 0. Therefore, β = 10 was selected to construct the hierarchical clustering tree (Supplementary Figure S3). The hierarchical clustering dendrogram revealed that each tree branch formed a distinct co-expression module, with each leaf representing a gene (Figure 5A). After preprocessing the expression data, a total of 21,960 genes were included in the WGCNA, resulting in the identification of 28 co-expression modules (Supplementary Table S6). Further, module–trait relationship analysis was used to evaluate the preservation of co-expression modules in *Lc3* and *Lc5* under varying shading conditions. The MEbrown module contained 2274 genes, the MEmidnightblue module had 407 genes, the MEpurple module consisted of 583 genes, the MEtan module contained 535 genes, and the MEred module contained 1218 genes (Supplementary Table S6). In most modules, gene expression trends between the two cultivars were nearly identical under different shading conditions, as shown in the MEpurple, MEtan, and MEred modules. However, two specific modules in *Lc3*—MEbrown (r = 0.94, *p* = 0.005) and MEmidnightblue (r = 0.83, *p* = 0.04)—exhibited distinct responses to shading treatments, suggesting that they are unique to the *Lc3* cultivar.

**Figure 6:**
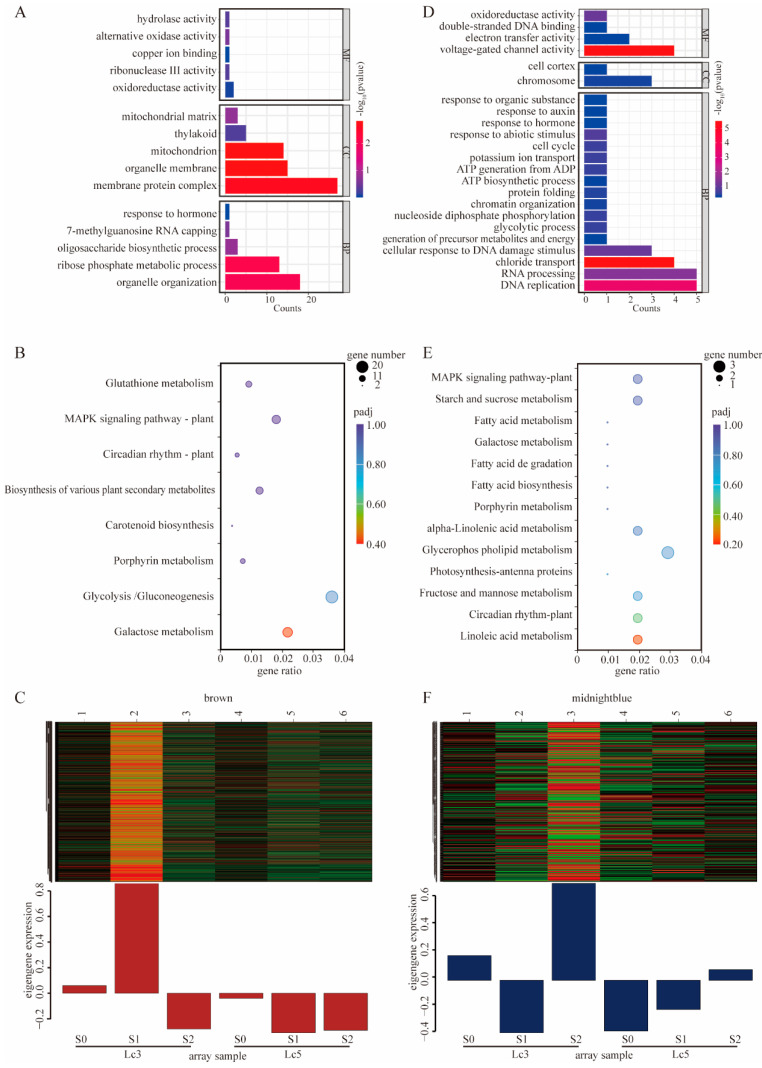
The GO and KEGG analyses of DEGs in the modules identified as MEbrown and MEmidnightblue through WGCNA. (**A**) The top 15 GO terms in the MEbrown module. (**B**) Significantly enriched KEGG pathways in the MEbrown module. (**C**) The expression of eigengenes for various treatments in the MEbrown module. The heatmap, ranging from red to green, indicates gene expression levels from high to low. (**D**) The top 23 GO terms in the MEmidnightblue module. (**E**) Significantly enriched KEGG pathways in the MEmidnightblue module. (**F**) The expression of eigengenes for various treatments in the MEmidnightblue module. The heatmap, ranging from red to green, indicates gene expression levels from high to low.

To better understand the biological significance of these modules, GO and KEGG pathway enrichment analyses were performed. In the MEbrown module, GO terms were enriched across the categories of BP, CC, and MF and included oxidoreductase activity, hormone response, the oligosaccharide biosynthetic process, and the mitochondrial matrix (Figure 6A). KEGG pathways were enriched in glycolysis/gluconeogenesis, galactose metabolism, and porphyrin metabolism (Figure 6B). Notably, the expression of key genes in the MEbrown module was most prominent in *Lc3*-S1 (Figure 6C). Similarly, the MEmidnightblue module, which was most active under the *Lc3*-S2 treatment, was enriched for GO terms related to oxidoreductase activity, response to abiotic stimulus, and ATP generation from ADP (Figure 6D,F). The key KEGG pathways in this module included starch and sucrose metabolism and galactose metabolism (Figure 6E), with highly expressed genes contributing to metabolic flexibility under extreme shading conditions. Interestingly, the MEmidnightblue module, which was strongly associated with *Lc3*-S2, was highly enriched in starch and sucrose metabolism and galactose metabolism (Figure 4 and Figure 6B). The integration of the results of WGCNA and KEGG enrichment analysis identified four key metabolic pathways—starch and sucrose metabolism, glycolysis/gluconeogenesis, galactose metabolism, and flavonoid biosynthesis—as central to the shading stress response of *L. chinensis*. Thus, these results indicate that the KEGG metabolic pathways and genes in the brown and midnightblue modules play a crucial role in the resistance of *Lc3* to shading stress.

**Figure 7:**
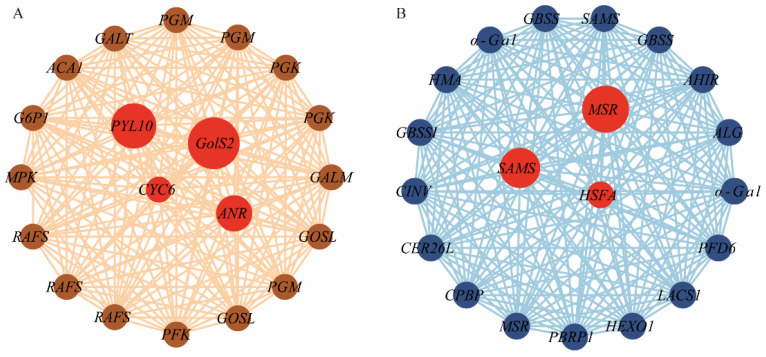
A visual analysis of gene co-expression correlations in the brown and midnightblue modules using Cytoscape. (**A**) Co-expression network of the brown module, highlighting the hub gene *LcGolS2* and its interactions with key genes in galactose metabolism (*RAFS*, *GALT*, *GALM*) and glycolysis/gluconeogenesis (*PGM*, *PGK*, *PFK*). (**B**) Co-expression network of the midnightblue module, focusing on genes involved in starch and sucrose metabolism (*GBSSI*, *GBSS*, *SUS*, *α-Gal*) and their regulatory relationships. Each node corresponds to a differentially expressed gene, with the edges between genes indicating co-expression relationships. The circle size reflects the level of connectivity.

The software Cytoscape was used to visualize the KEGG hub genes within the brown and midnightblue modules. In the brown module, *GolS2* (h2_2Ns000960) was identified as a hub gene, exhibiting co-expression correlations with pathways such as galactose metabolism that included *RAFS*, *GALT*, and *GALM* genes, and pathways such as glycolysis/gluconeogenesis that comprised *PGM*, *PGK*, and *PFK* genes (Figure 7A). In the midnightblue module, numerous interconnected genes that formed a complex regulatory network involving genes such as *GBSSI*, *GBSS*, *SUS*, and *α-Gal* were identified (Figure 7B). The analysis revealed a strong relationship between genes associated with starch and sucrose metabolism and galactose metabolism pathways (Figure 7B). Furthermore, the hub gene *GolS2* in the brown module suggested a previously unknown role, which indicates its potential as a target for future research.

### 2.6. The Validation of the Expression Levels of Genes Related to Different Pathways Using qRT-PCR

**Figure 8:**
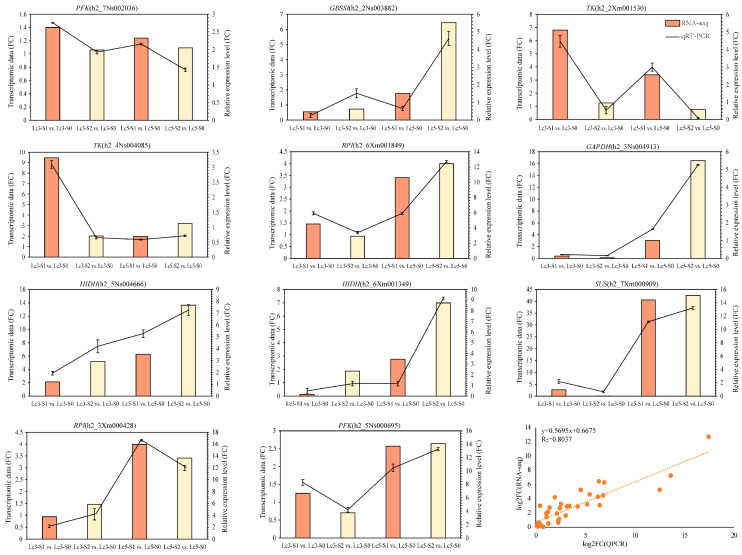
The RNA-seq data was validated by quantitative real-time PCR.

To further confirm the RNA-Seq results, qRT-PCR was used to verify the changes in the expression of genes related to starch and sucrose metabolism, glycolysis/gluconeogenesis, galactose metabolism, and flavonoid biosynthesis under shading stress. Although the fragments per kilobase of transcript per million mapped reads (FPKM) values of the selected genes and the relative quantitative values of qRT-PCR differed, the trends in gene expression were consistent (Figure 8). The qRT-PCR results of *LcPFK* (h2_7Ns002036, h2_5Ns000695), *LcGBSSI* (h2_2Ns003882), *LcTK* (h2_2Xm001530, h2_4Ns004085), *LcRpi* (h2_6Xm001849, h2_3Xm000428), *GAPDH* (h2_3Ns004913), *HIDH* (h2_5Ns004666, h2_6Xm001349), and *LcSUS* (h2_6Xm001349) were highly consistent with the FPKM values from RNA-Seq. These results further demonstrate that the difference in resistance between *Lc3* and *Lc5* cultivars in response to shade stress is highly correlated with structural genes related to different metabolic pathways.

## 3. Discussion

In this study, *L. chinensis* cultivars Zhongke No. 3 (*Lc3*) and Zhongke No. 5 (*Lc5*), which differ in shade tolerance, were subjected to different levels of shading stress. This study used physiological measurements and transcriptomic analysis to elucidate the physiological and molecular mechanisms underlying the differences in shading stress tolerance between the two genotypes. These findings provide new insights into the plant’s adaptive strategies under shading stress.

### 3.1. Physiology Responses of Lc3 and Lc5 to Different Shade Treatments Were Different

Shade is an abiotic stress prevalent in crop production that significantly impacts plant growth and development [30]. In this study, varying levels of shading stress significantly inhibited the growth of *Lc3* and *Lc5*, with the two genotypes displaying distinct morphological responses as the intensity of shading increased (Figure 1A). The results suggest that *Lc3* exhibits greater adaptability to shading stress, with its healthy growth conditions and enhanced physiological traits providing critical support for its resistance to shading stress. Additionally, both S1 and S2 shading types reduced plant height and caused necrosis of the *L. chinensis* leaves, suggesting that the disruption of the chlorophyll structure by shading stress led to decreased chlorophyll content and reduced photosynthetic activity [13,31]. These results were also supported by a reduction in photosynthetic parameters such as *Pn*, *Gs*, and *Tr* under shading stress, with Lc5 more severely affected (Figure 1C).

Shading stress also damages the selective permeability of the cell membrane, thus altering the dynamic balance of ions and the efflux of soluble substances. Plants can mitigate this stress-induced damage by increasing their levels of osmotic regulators and antioxidant enzyme activity [32]. Osmotic regulators such as flavonoids, carotenoids, AsA, and glutathione have antioxidant and free radical scavenging properties that help plant cells to regulate osmotic pressure and cope with environmental stress [19,27]. Abiotic stress often leads to the excessive accumulation of ROS within the cells. In this study, as the shading intensity increased, *Lc3* exhibited higher POD and SOD activity but lower MDA content than *Lc5* in both S1 and S2 treatments, indicating a large amount of ROS accumulation and severe oxidative damage in *Lc5* (Figure 1D). The results also suggest the stronger capacity of *Lc3* to scavenge ROS under shading stress and thereby limit damage to its cell membrane, and demonstrate its greater resistance to shading stress.

Shading stress in plants encompasses several physiological events, including the inhibition of soluble sugars such as sucrose and fructose, delayed starch degradation and consumption, and the disruption of flavonoid secondary metabolism, which is a complex process regulated by multiple genes and pathways [11,12]. Transcriptomics, employed to further investigate the mechanisms and key pathways associated with shade tolerance in *Lc3* and *Lc5* cultivars, revealed the significant enrichment of DEGs in both cultivars. This occurred in the starch and sucrose metabolism and glycolysis/gluconeogenesis pathways, indicating that these pathways are crucial for plant growth in response to shading stress. Nevertheless, under shading stress, *Lc3* cultivars exhibited significant enrichment in the starch and sucrose metabolism, flavonoid biosynthesis, and galactose metabolism pathways (Figure 4C,F), which may account for its shade tolerance.

### 3.2. Saccharide-Related Pathway in Response to Shade Stresses

**Figure 9:**
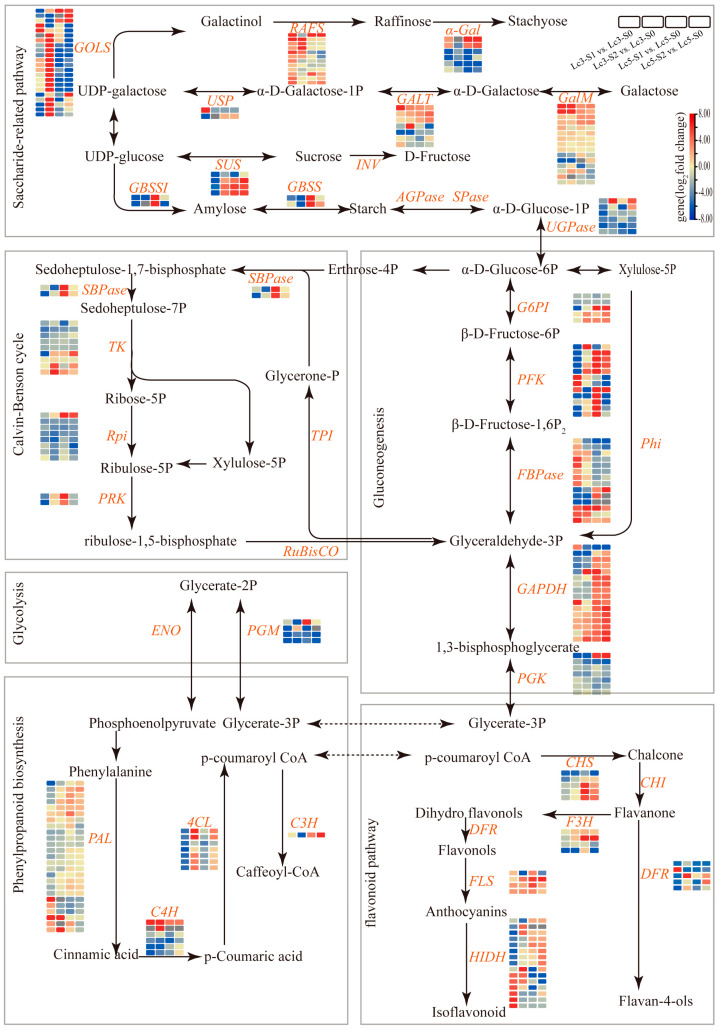
A hypothetical model of the response mechanisms of resistant and sensitive species under shading stress. Red and blue in this model indicate upregulated and downregulated genes under shading stress, respectively. Orange letters represent genes coding for enzymes. *GOLS*, galactinol synthase; *RAFS*, raffinose synthase; *α-Gal*, alpha-galactosidase; *GALT*, galactose-1-phosphate uridylyltransferase; *GalM*, aldose 1-epimerase; *USP*, UDP-sugar pyrophosphorylase; *SUS*, sucrose synthase; *GBSSI*, granule-bound starch synthase I; *GBSS*, granule-bound starch synthase; *INV*, invertase; *AGPase*, ADP-glucose pyrophosphorylase; *SPase*, starch phosphorylase; *UGPase*, UDP-glucose pyrophosphorylase; *SBPase*, sedoheptulose-1,7-bisphosphatase; *TK*, transketolase; *Rpi*, ribose-5-phosphate isomerase; *PRK*, phosphoribulokinase; *RuBisCO*, ribulose-1,5-bisphosphate carboxylase/oxygenase; *TPI*, triose-phosphate isomerase; *G6PI*, glucose-6-phosphate isomerase; *PFK*, phosphofructokinase; *FBPase*, fructose-1,6-bisphosphatase; *GAPDH*, glyceraldehyde-3-phosphate dehydrogenase; *PGK*, phosphoglycerate kinase; *PGM*, phosphoglycerate mutase; *ENO*, enolase; *PAL*, phenylalanine ammonia-lyase; *4CL*, 4-coumarate-CoA ligase; *C4H*, cinnamate 4-hydroxylase; *C3H*, p-coumaroyl shikimate 3-hydroxylase; *CHS*, chalcone synthase; *CHI*, chalcone isomerase; *F3H*, flavanone 3-hydroxylase; *DFR*, dihydroflavonol-4-reductase; *FLS*, flavonol synthase; *HIDH*, isoflavone reductase.

Carbohydrates are the primary energy source for plant cells and also function as critical signaling molecules that regulate plant responses to various stresses, including salinity, alkalinity, and shading [33]. Under shading stress, plants experience changes in the accumulation of soluble sugars such as sucrose, fructose, and galactose, which modulate osmotic balance, regulate physiological processes, and help mitigate stress-induced damage [34]. In this study, functional enrichment analysis of DEGs indicated that most carbohydrate-related genes were induced, suggesting that carbohydrates may be related to the different responses of the two cultivars to shading stress (Figure 9, Supplementary Table S7). Among these genes, those encoding galactinol *GolS*, a key enzyme in galactose metabolism, were upregulated in both *Lc3* and *Lc5* in response to shading stress. However, the shade-tolerant genotype *Lc3* exhibited significantly higher expression of the *LcGolS* gene compared to *Lc5* (Figure 9). Galactinol, a product of *GolS* activity, regulates the synthesis of raffinose and stachyose, which serve as osmoprotectants to enhance stress tolerance [35,36]. Previous studies have shown that photoperiods can strongly induce *GolS* expression in cucumber (*Cucumis sativus* L.), while overexpression of the soybean *GmGolS2-2* gene in tobacco improves abiotic stress tolerance by increasing soluble sugar content [37]. Therefore, the upregulation of *GolS* genes in *Lc3* in this study suggests that galactinol metabolism plays a pivotal role in enhancing the shade tolerance of *L. chinensis* (Figure 9, Supplementary Table S7). Galactitol, a derivative of galactinol, also aids in ROS clearance and acts as a signaling molecule under abiotic stress in *Arabidopsis* [38,39]. Therefore, *Lc3* likely accumulates higher levels of galactitol under S1 and is thereby conferred superior resistance to shading stress compared to *Lc5*.

The genes involved in the starch and glycolysis/gluconeogenesis pathways also displayed distinct expression patterns between the *Lc3* and *Lc5* cultivars. In *Lc3*, genes encoding phosphofructokinase (*PFK*) and fructose-1,6-bisphosphatase (*FBPase*), key enzymes in glycolysis and gluconeogenesis, were significantly upregulated. *PFK* serves as a rate-limiting enzyme in glycolysis, while *FBPase* regulates carbon flux between sugar breakdown and synthesis. The overexpression of the *MdPFK5* gene in Arabidopsis and apple enhances salt tolerance, partly through soluble sugar accumulation [40], while the expression of *FBPase* alleviates photosynthetic limitations under low-nitrogen conditions in soybeans [41]. Therefore, the upregulation of *PFK* and *FBPase* in *Lc3* supports energy production and carbohydrate utilization, contributing to its shade tolerance.

The *PGM* and *PGK* genes were also significantly expressed in *Lc5* but not in *Lc3* (Figure 9). *PGM* converts glucose-1-phosphate to glucose-6-phosphate, which feeds into glycolysis, while *PGK* generates ATP. The expression pattern of these genes in *Lc5* is consistent with significant changes in the activity of glycolytic genes in ageing cells [42], indicating that *Lc5* compensates for energy deficiencies caused by shade with an increased reliance on glycolysis and carbohydrate reserves. In contrast, *Lc3* reduces its dependence on glycolysis when under shading stress by enhancing its photosynthetic efficiency and activating non-sugar metabolic pathways, such as fatty acid oxidation. These findings underscore the importance of carbohydrate-related metabolic pathways and gene regulation in determining the differential shading stress responses of *L. chinensis* genotypes.

### 3.3. The Flavonoid Biosynthesis Pathway Positively Responds to Shade Stresses in L. chinensis

Flavonoids are crucial secondary metabolites produced by plants in response to various environmental stresses, including shading stress, and are present in a wide range of plants. Flavonoids exhibit antioxidant, anti-inflammatory, and antibacterial activity effects and effectively neutralize excessive ROS, thereby reducing oxidative damage and improving plant survival and adaptability under environmental stress [43]. In this study, the flavonoid metabolic pathway was significantly enriched in *Lc3* and *Lc5*, highlighting its crucial role in plant responses to shading stress. Flavonoids, which exhibit significant protective effects under shading stress, are strongly influenced by shading conditions, which modulate their synthesis [44,45,46,47]. However, shading stress significantly modulated higher expression of flavonoid-related genes in *Lc3* than in *Lc5* (Figure 9), which likely enhanced the antioxidant capacity of *Lc3* plants and thereby aided their adaptation to environmental stress. Several key genes such as *PAL*, *4CL*, *CHS*, *CHI*, *F3H*, and *FLS* play a central role in the flavonoid biosynthesis pathway [48]. Among the 30 structural genes annotated in this pathway, all the structural genes of *PAL* enzymes, including *LcPAL1*, *LcPAL2*, and *LcPALY*, were upregulated in *Lc3*, while most were downregulated in *Lc5*. The expression levels of *C4H*, *4CL*, and *HIDH* genes were also significantly higher in *Lc3* than in *Lc5* (Figure 9). These findings underscore the role of shading stress in enhancing flavonoid accumulation, which helps maintain ROS balance and improves plants’ stress tolerance and adaptation to environmental stress [49,50]. Overall, this study provides novel insights into the molecular mechanisms of flavonoid stress adaptation and identifies key genetic targets for developing stress-resistant plant cultivars through breeding programs.

## 4. Conclusions

**Figure 10:**
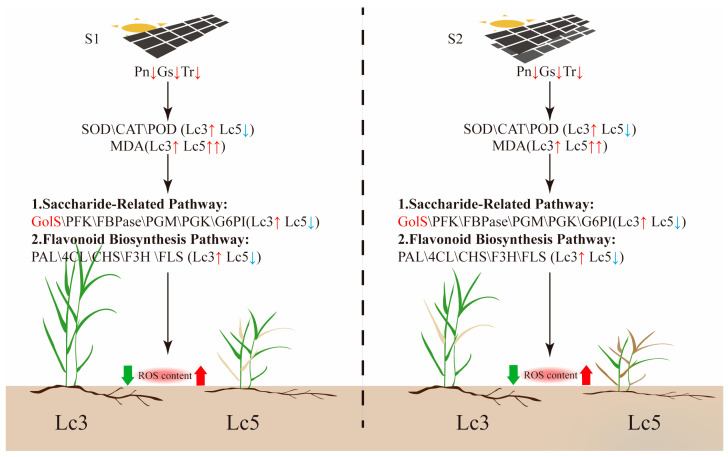
A hypothetical model of the response mechanisms of shade-tolerant (*Lc3*) and shade-intolerant (*Lc5*) genotypes under shading stress. S1 represents single-layer shading, and S2 represents double-layer shading.

The physiology and molecular mechanisms underlying plant responses to shading stress are intricate and multifaceted. In this study, a combination of physiological and transcriptomic approaches were used to explore the key regulatory pathways and adaptive mechanisms between the shade-tolerant *Lc3* and the shade-intolerant *Lc5* genotypes under shading stress. Transcriptomic analysis identified five regulatory pathways associated with the growth of *L. chinensis* under shading stress, with the starch and sucrose metabolism and glycolysis/gluconeogenesis pathways consistently and significantly enriched in all S1 and S2 shaded plants. However, the starch and sucrose metabolism, flavonoid biosynthesis, and galactose metabolism pathways were prominently enriched in *Lc3* and supported its superior shade tolerance due to the pathways alongside the associated DEGs. The superior shade tolerance in *Lc3* is also supported by its relatively greater plant height, photosynthetic rate, and antioxidant capacity compared to *Lc5*. The shading stress also significantly altered the expression of *GolS*, *PFK*, *FBPase*, *PGM*, *PGK*, *G6PI*, *GAPDH*, *PAL*, and *4CL* genes, many of which are involved in starch and sucrose metabolism and flavonoid biosynthesis (Figure 10). Therefore, the growth of *L. chinensis* under shading stress is regulated by coordinated changes in starch and sucrose metabolism, glycolysis/gluconeogenesis, flavonoid biosynthesis, and galactose metabolism. These findings provide valuable insights into the regulatory mechanisms of plant responses to shading stress and lay the groundwork for molecular breeding strategies aimed at developing shade-tolerant crops like *L. chinensis*.

## 5. Materials and Methods

### 5.1. Plant Materials and Treatments

The shading experiments were conducted in a greenhouse at Qingdao Agricultural University, Qingdao, China, using *L. chinensis* plants with a uniform height of 10 cm at the start of the experiment. The pots used in the experiment had a diameter of 14 cm and a height of 10 cm. The substrate consisted of a mixture of loam, vermiculite, and perlite in a ratio of 3:2:1. To simulate shading under photovoltaic panels, shade nets with either single-layer shading (S1) or double-layer shading (S2) were placed approximately 2 m above the plants, with the control plants only exposed to natural light (S0). Standard agricultural practices for fertilization, irrigation, and pest control were applied uniformly throughout the experiment. After 30 days of shading treatment, leaf samples were collected from the tops of the plants in triplicates, immediately frozen in liquid nitrogen, and then stored at −80 °C for subsequent physiological and transcriptomic analyses.

### 5.2. Measurement of Growth and Morphological Characterization and Determination of Chlorophyll Content

After 30 days of exposure to shading, plant growth parameters, including plant height, chlorophyll content, relative conductivity, and relative water content, were measured. To determine chlorophyll content, 0.1 g of fresh *L. chinensis* leaves were cut into small pieces and immersed in 10 mL of acetone for overnight extraction until the leaves were completely white. The resulting extract was thoroughly mixed and analyzed using a UV spectrophotometer (SHIMADZU UV-2700, Kyoto, Japan) at wavelengths of 663 nm and 646 nm [51]. Chlorophyll content was determined using the following formula: chlorophyll a = 12.21A_663_ − 2.81A_646_, chlorophyll b = 20.13A_646_ − 5.03A_663,_ where A is the absorbance at the specific wavelengths.

### 5.3. Measurements of Photosynthetic Parameters and Antioxidant Enzyme Activities

The photosynthetic parameters, including Pn, Gs, Ci, and E, were measured using a Li-6800 photosynthetic system (LI-COR, Lincoln, NE, USA) on the second fully expanded *L. chinensis* leaf from the apex between 9:00 a.m. and 12:00 p.m. on a sunny day in triplicates for each leaf. The activities of SOD, CAT, POD, and MDA from the control and treated *L. chinensis* leaves were measured using commercial kits supplied by Suzhou Keming Biotechnology Co. (Suzhou, China), according to the manufacturer’s instructions.

### 5.4. Transcriptome Analysis and Quantitative Real-Time PCR (qRT-PCR) Validation

The total RNA was extracted from *L. chinensis* leaves using the FastPure Universal Plant Total RNA Isolation Kit (VAZYME, Nanjing, China), according to the manufacturer’s instructions. The cDNA was obtained using the HiScript IV 1st Strand cDNA Synthesis Kit (+gDNA wiper) (RC411-01; VAZYME, Nanjing, China), according to the manufacturer’s instructions. The cDNA libraries for the 18 *L. chinensis* leaf samples were constructed and sequenced using the Illumina Novaseq6000 platform (BGI Bioinformatics Institute, Beijing, China). Specifically, 18 cDNA libraries were generated, with 9 libraries derived from *Lc3* (3 for each shading treatment: S0, S1, and S2) and 9 from *Lc5* (3 for each shading treatment: S0, S1, and S2). The raw sequence data were processed by filtering out low-quality reads. Fastp v 0.19.3 was used to filter raw data by mainly removing reads containing adapters. Specifically, sequencing error rates and GC content distribution were assessed to ensure the acquisition of clean reads suitable for downstream analyses. StringTie v1.3.4d was used for novel gene prediction and de novo assembly of clean reads. The genes were annotated by aligning sequences with those found in the *Non-Redundant* (NR), *Nucleotide* (NT), *Kyoto Encyclopedia of Genes and Genomes* (KEGG), *Swiss-Prot*, *PFAM*, GO, and *Clusters of Orthologous Groups* (KOG) databases. The differential gene expression analysis between two groups of the different shading groups S0, S1, and S2 was conducted using the DESeq2 v1.22.1 R package, while the ClusterProfiler v4.14.6 R package was used to investigate the enriched DEGs in KEGG pathways. The criteria for identifying differential genes included the use of |log_2_FoldChange| > 1 and FDR < 0.05. The qRT-PCR was performed on a Bio-RAD CFX96™ Touch (Bio-Rad Laboratories, Inc., Hercules, CA, USA) using ChamQ SYBR Color qPCR Master Mix (VAZYME, Nanjing, China), with Actin as an internal reference primer. The qRT-PCR reaction mixture was carried in a total of 20 µL consisting of 10 µL ChamQ SYBR Green qPCR Master Mix (Q411, Vazyme, Nanjing, China), 0.4 µL of each forward and reverse primer, and 2 µL of cDNA. The amplification program consisted of an initial denaturation at 95 °C for 30 s followed by 39 cycles, each consisting of denaturation at 95 °C for 10 s; annealing at 60 °C for 30 s; and extension at 72 °C for 30 s. All primers used in this study are listed in Table S8.

### 5.5. WGCNA

Based on previous studies [52], the minimum appropriate sample size was employed for the weighted correlation network analysis. A threshold of FPKM < 1 was applied to filter out invalid gene expressions in each sample. The R software package v4.14.6 was used to automate the network to construct co-expression modules comprising the selected genes. Subsequently, adjacency matrices were converted into topological overlap matrices to identify modules associated with specific *L. chinensis* samples in the reconstructed network. Genes with similar expression patterns were grouped into the same module and used to identify hub genes. The eigengene values of each module were calculated and used to analyze the correlation between genes and traits. Subsequently, Cytoscape v.3.10.2 software was used to visualize the co-expression networks.

### 5.6. Statistical Analysis

All data analyses and visualizations were performed using GraphPad Prism 8.0 (GraphPad Software, Boston, MA, USA). The effects of different shading treatments on physiological parameters were evaluated using SPSS 19.0 (IBM Corp., New York, NY, USA). Statistical significance was evaluated using one-way analysis of variance (ANOVA), followed by Tukey’s post hoc test and Student’s *t*-test, with significance levels defined as * *p*  <  0.05 and ** *p * <  0.01. DEMs and DEGs were represented as heat maps and generated through TBtools v2.012.

## Data Availability

Data is contained within the article or Supplementary Materials.

## Supplementary Materials

The following supporting information can be downloaded at: https://www.mdpi.com/article/10.3390/ijms26062730/s1.
