# Supplementary material for: The Combination of Physiological and Transcriptomic Approaches Reveals New Insights into the Molecular Mechanisms of Leymus chinensis Growth Under Different Shading Intensities
Source: Int J Mol Sci. 2025 Mar 18;26(6):2730. doi: 10.3390/ijms26062730 (PMC11942481; doi:10.3390/ijms26062730)
Supplement: Supplementary file 1 [file ijms-26-02730-s001.zip › Supplementary Figure S3.pdf]

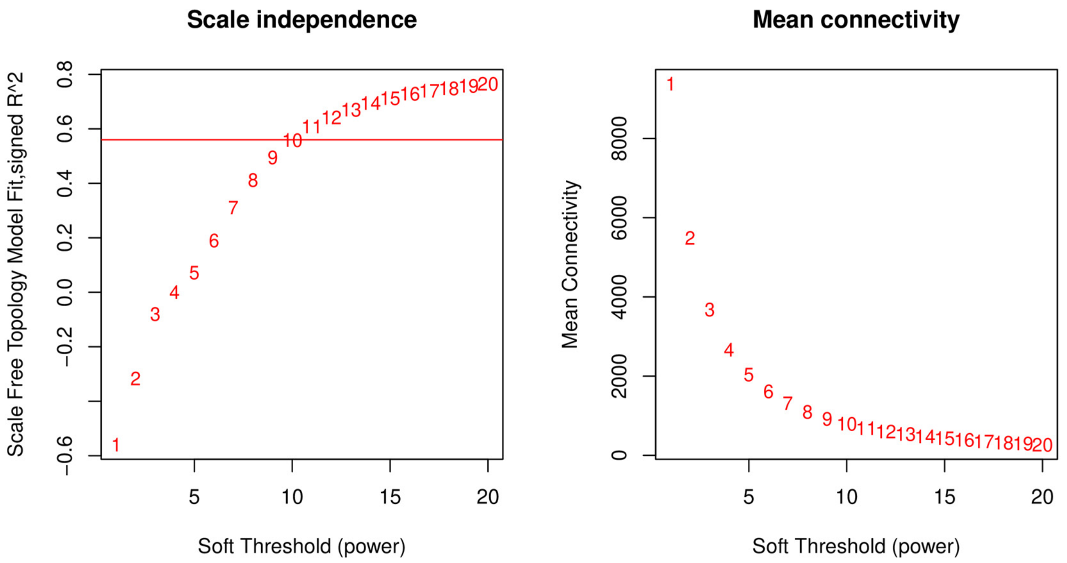

Supplementary Figure S3： Analysis of network topology for various soft-thresholding powers. The left panel shows the scale-free fit index (y-axis) as a function of the soft-thresholding power (x-axis). Power 10 was chosen because the fit index curve flattened out upon reaching a high value (> 0.6). The right panel displays the mean connectivity (degree, y-axis) as a function of the soft-thresholding power (x-axis).
