# Supplementary figures and images for: The Combination of Physiological and Transcriptomic Approaches Reveals New Insights into the Molecular Mechanisms of Leymus chinensis Growth Under Different Shading Intensities
Source: Int J Mol Sci. 2025 Mar 18;26(6):2730. doi: 10.3390/ijms26062730 (PMC11942481; doi:10.3390/ijms26062730)

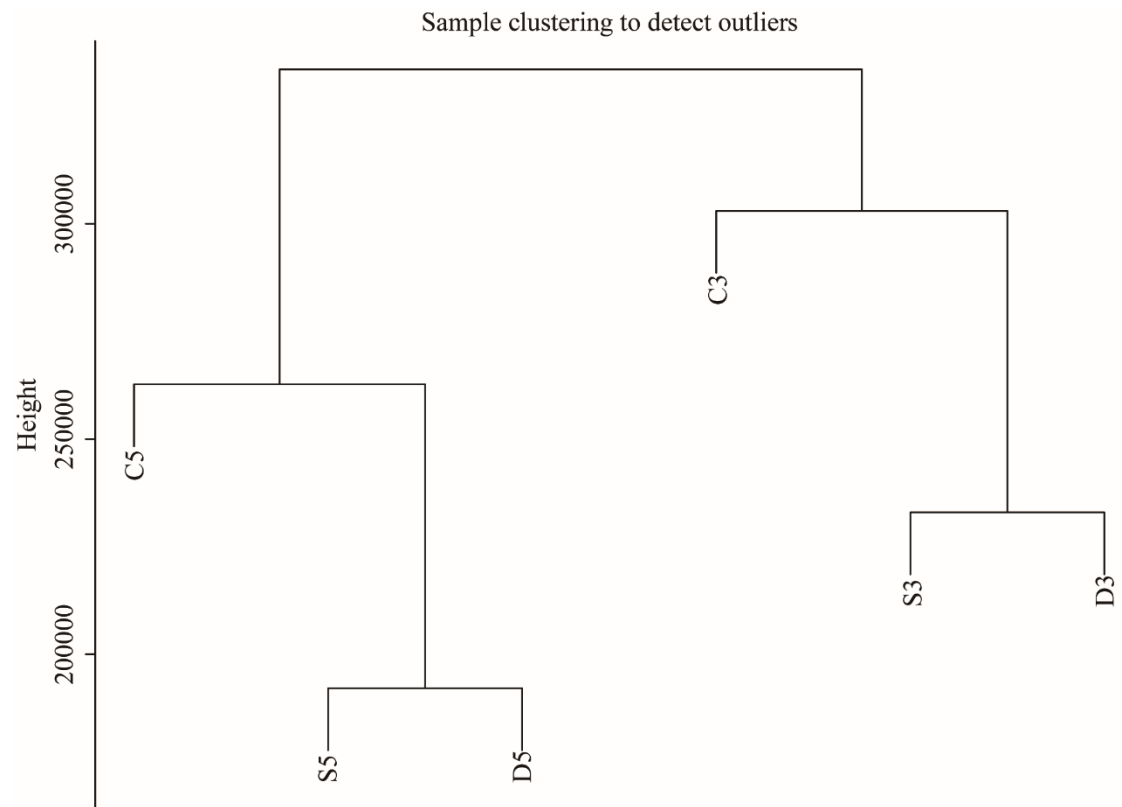

Supplementary Figure S2: Sample clustering found no obvious outliers.

Supplement: Supplementary file 1 [file ijms-26-02730-s001.zip › Supplementary Figure S2.pdf]
